# Single 3.3 fs multiple plate compression light source in ultrafast transient absorption spectroscopy

**DOI:** 10.1038/s41598-021-92102-5

**Published:** 2021-06-18

**Authors:** Ronnie R. Tamming, Chao-Yang Lin, Justin M. Hodgkiss, Shang-Da Yang, Kai Chen, Chih-Hsuan Lu

**Affiliations:** 1 School of Chemical and Physical Sciences, Victoria University of Wellington, Wellington, 6012 New Zealand; 2 MacDiarmid Institute for Advanced Materials and Nanotechnology, Wellington, 6012 New Zealand; 3 Institute of Photonics Technologies, National Tsing Hua University, Hsinchu, 30013 Taiwan; 4 Robinson Research Institute, Faculty of Engineering, Victoria University of Wellington, Wellington, 6012 New Zealand; 5 Wellington UniVentures, Victoria University of Wellington, Wellington, 6012 New Zealand; 6 The Dodd-Walls Centre for Photonic and Quantum Technologies, Dunedin, 9016 New Zealand

**Keywords:** Chemistry, Materials science, Optics and photonics, Physics

## Abstract

Ultrafast transient absorption spectroscopy is a powerful tool to reveal excited state dynamics in various materials. Conventionally, probe pulses are generated via bulk supercontinuum generation or (noncollinear) optical parametric amplifiers whilst pump pulses are generated separately using (noncollinear) optical parametric amplifiers. These systems are limited by either their spectral density, stability, spectral range, and/or temporal compressibility. Recently, a new intense broadband light source is being developed, the multi-plate compression, which promises to overcome these limitations. In this paper, we analyze the supercontinuum generated by a single Multiple Plate Compression system to set a benchmark for its use in the field of ultrafast pump-probe spectroscopy. We have compressed the supercontinuum to 3.3 fs using chirp mirrors alone, making it an excellent candidate for pump-probe experiments requiring high temporal resolution. Furthermore, the single light source can be used to generate both probe and pump pulses due to its high spectral density (>14.5 nJ/nm) between 490 and 890 nm. The intensity has an average shot-to-shot relative standard deviation of 4.6 % over 490 to 890 nm, calculated over 2,000 sequential shots. By using only 1,000 shot pairs, a $\Delta T/T$ noise level of $2.6\times 10^{-4}$ RMS is achieved. Finally, as a proof of concept, the transient absorption spectrum of a methylammonium lead iodide perovskite film is taken, showing great signal to noise with only 1,000 shot pairs. These results show great potential for the employment of this technique in other spectroscopic techniques such as coherent multidimensional spectroscopy.

## Introduction

Transient Absorption Spectroscopy (TAS) is an often used and powerful tool to examine excited state dynamics in materials such as charge transfer^1^, charge and exciton diffusion^2,3^ and hot carrier cooling^4,5^. This spectroscopic technique utilizes a probe and a pump pulse, overlapping at the sample position. The pump generates an excited state population and a second pulse probes the sample by way of transmission. For TAS, a stable broadband probe is required to obtain a full picture of the excited state on sub-percentage modulation levels^6^. As for the pump, a stable intense pulse (μJ level) is preferred. There are two opposing spectral requirements for different areas of interest. The first option is a compressed broadband pump to obtain high temporal resolution, whilst the second option is a wavelength tuneable narrowband excitation to resonate with specific electronic transitions. To account for the different requirements, a combination of the available light sources is currently used to generate these different pulses.

Several light sources that have been used are bulk supercontinuum generation^6,7^, nonlinear optical fibers (such as hollow core fibers)^8–10^, and (Noncollinear) Optical Parametric Amplifiers ((N)OPA)^6,11^. Because of the different properties of these light sources, they are generally used for specific parts of the TAS experiment. Therefore, a combination of these methods is required, adding to the complexity of this technique. The bulk supercontinuum can result in more than an octave-spanning spectrum as it relies on the high nonlinear coefficient of solid-state media^12^. However, the input pulse energy is limited to the J level, beyond which optical damage will occur within the bulk crystal^12,13^, resulting in an overall low spectral density in the 10 pJ/nm range, which makes it unsuited as a pump. Hollow core fibers overcome the damage threshold by generating a supercontinuum in a gas phase^14^. This results in a broad and bright spectrum, however, there are various competing nonlinear processes present in these fibers, such as the chaotic four-wave mixing and modulation instability^9,15–17^. These mechanisms, combined with their sensitive input coupling, result in strong spectral fluctuations of the generated supercontinuum. (N)OPAs, on the other hand, are stable and intense light sources. Their limitation is the intrinsic spatial chirp of the output beam and the available spectral range due to different phase matching conditions for different parts of the spectrum. The latter can only be overcome by implementing complex schemes^18,19^, that would add to a multitude of different optical components already required for the (N)OPA itself^20,21^.

In this paper, we will demonstrate the feasibility of using a novel single light source, the Multiple Plate Compression (MPC), in the field of ultrafast pump-probe spectroscopy. This MPC system, where pulses are focused onto a series of thin plates, makes use of the high nonlinear coefficient of solid-state materials while avoiding destructive mechanisms. It has the capability of generating octave spanning supercontinuum pulses with a high spectral density (> 9.5 nJ/nm) in the visible to NIR^22,23^. These pulses allow for temporal compression, down to single cycle, using only chirped mirrors^24^. The system requires little to no daily alignment and provides long-term stability required for spectroscopic measurements with weak signal levels. On top of this, the high photon count allows for both the pump and probe to be generated by a single MPC setup, vastly reducing the experimental complexity of the TAS system.

## Methods

**Figure 1:**
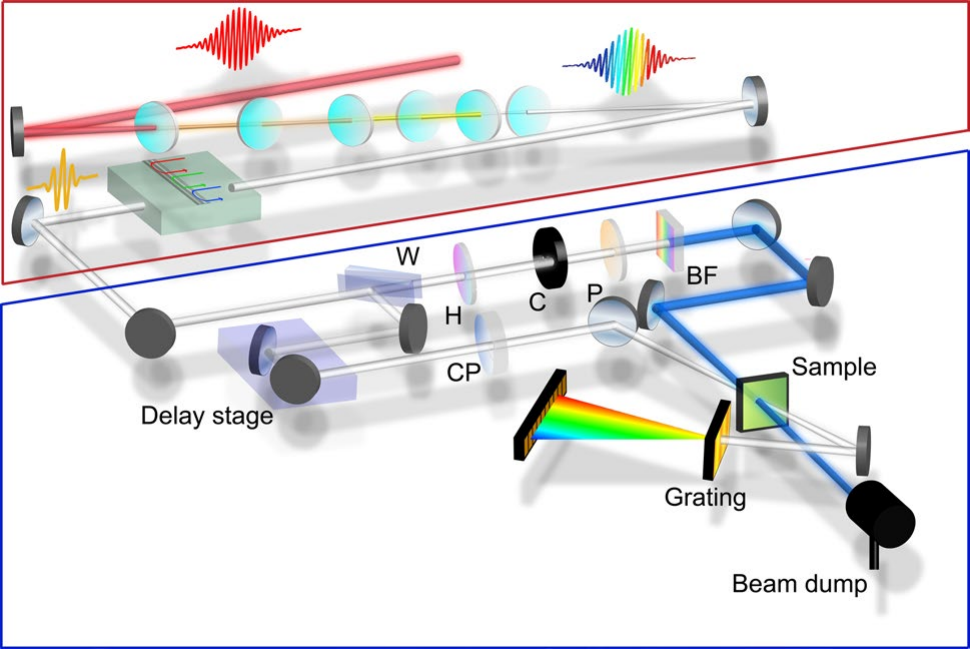
Scheme of the MPC-TAS system used in this paper, with the MPC part indicated by the red box and the TAS part indicated by the blue box. CM, chirped mirrors; W, wedge pair; H, half-wave plate; C, chopper; P, wire-grid polarizer; SF, short-pass filter; CP, compensation plates

The MPC TAS scheme used for this paper is depicted in Fig. 1^25^. This setup consists of two main components; the first component, indicated by the red box, is the MPC supercontinuum source. A 1 kHz Ti:Sapphire amplifier system (Femtopower$^{{\mathrm{TM}}}$ HE PRO CEP) is used to generate the 25 fs fundamental pulses at 790 nm. The output of the amplifier is tuned to 200 μJ per pulse which results in an estimated peak intensity of 20 TW/cm$^2$ at the first plate. The beam passes through a total of 6 thin z-cut quartz plates (50 μm thick), set at Brewster’s angle. These plates are placed near the focal point as previously described by Lu *et al.*^22^ to obtain large spectral broadening (450 nm to 980 nm) while maintaining long-term stability.

After the supercontinuum generation, a set of commercially available chirped mirrors (DCM9, Laser Quantum) are used to compensate for linear dispersion (− 720 fs$^2$ GDD) while a pair of custom chirped mirrors are designed and used to compensate for the higher order dispersion. These custom chirped mirrors allow for compression of the pulse to sub-5 fs and suppress the intensity of the side lobes of the pulse in the temporal domain. These chirped mirrors lack the capabilities of polarization tuning, line-by-line shaping or precise waveform generation when comparing to pulse shapers, however, these mirrors are favoured for this application as they are compatible with the high pulse power, have a high throughput, are straightforward to align and can make use of the high beam quality and repeatable spectrum and dispersion of the pulses. The MPC supercontinuum has a total energy of 87 μJ per pulse, resulting in a 44% conversion efficiency. The conversion efficiency can be improved in the future as the main loss of this system is the limited spectral range of the on-shelf chirped mirrors.

**Figure 2:**
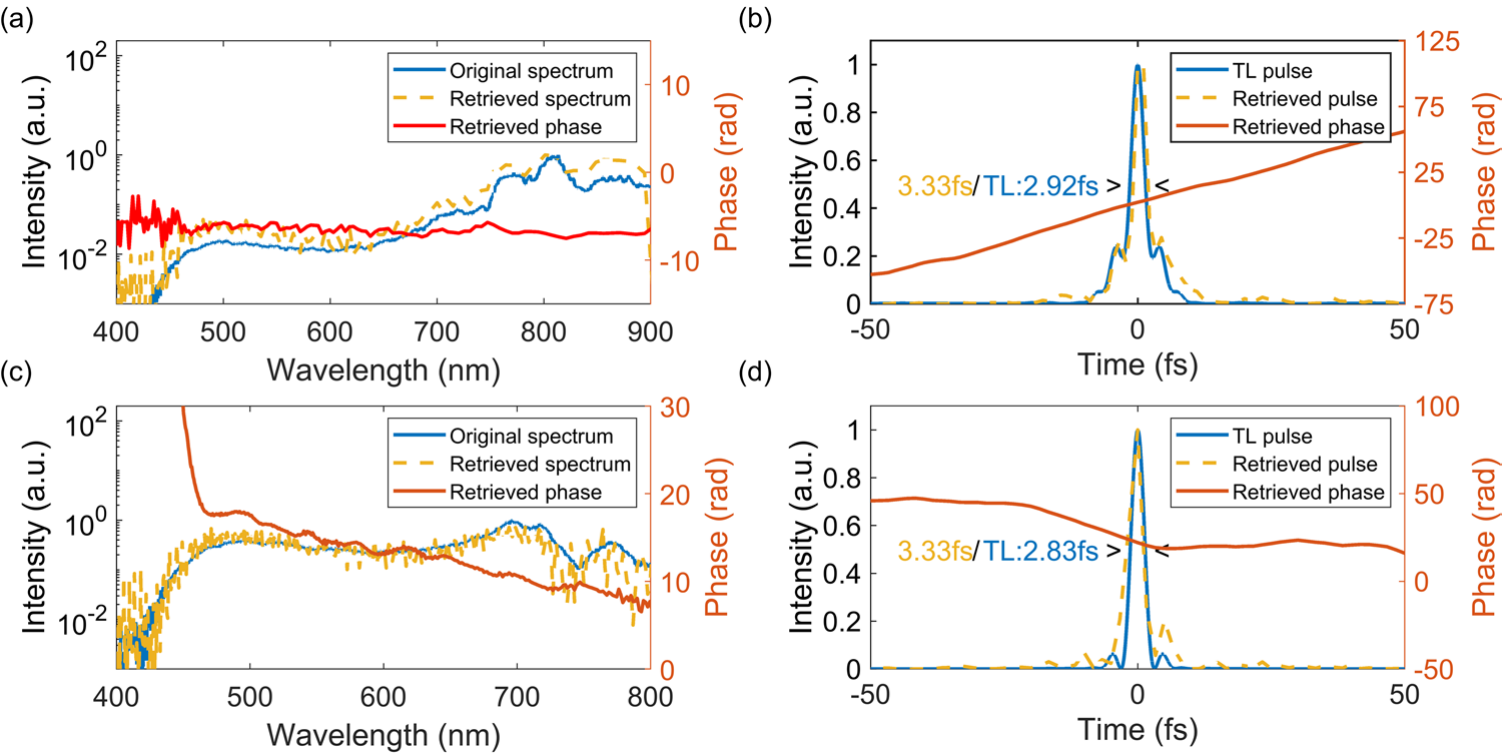
PG-XFROG measurement result of probe and pump pulse at the sample position. with (**a**) probe and (**c**) pump spectra directly recorded from the spectrometer (blue) and spectrum reconstructed from PG-XFROG trace (dashed orange) with phase (red). (**b**) and (**d**) are the reconstructed temporal intensity (dashed orange) and phase (red) of probe and pump pulse, respectively. The transform limit (blue) is obtained by directly using the Fourier transformation on the spectrometer data

The blue box in Fig. 1 contains the second part (TAS) of the system. The supercontinuum is split into a pump and probe beam by using the transmission and first Fresnel reflection of a wedge pair. For the pump (transmitted beam), a half-wave plate and wire-grid polarizer are used to attenuate the excitation energy and set the polarization of the pump. A chopper is used at half the laser output frequency to enable the measurement of sequential ground state and excited state shots. The indexing of the shots is done by a photodiode (not shown) which captures the back reflected part of the pump pulse from the wire-grid polarizer, placed at a small angle. The strong fundamental wavelength in the pump is filtered out by a 750 nm short-pass filter, resulting in a different spectrum for the probe and pump pulse (Fig. 2a,c). A concave mirror is then used to focus to a spot diameter of 500 μm (FWHM) at the sample.

The probe (first Fresnel reflection) is sent to a mechanical delay stage, to control the time delay between pump and probe. A compensation plate is placed in the probe path to add the same amount of dispersion to the probe path as the pump path. The beam is then focused onto the sample with a spot diameter of 100 μm (FWHM), where the ratio between pump and probe spot sizes ensures that the probe sees a uniform excitation profile. The probe is then collimated and focused onto a slit of a grating spectrometer (SpectraPro 2150, Princeton Instruments). All individual shots are collected by a fast photodiode array (Glaz Linescan II, Synertronic with S11639-01 CMOS, Hamamatsu). The optical configuration result in a spectral resolution of 0.27 nm/pixel.

## Results and discussion

The key parameter for high temporal resolution of the TAS system are short pulse durations, allowing for observations of faster kinetics. The MPC’s broadband and coherent nature has shown temporal compression down to several femtoseconds^24^. The pump and probe pulse durations are measured after the compression stage with a home-built PG-XFROG apparatus (see Additional methods). The obtained spectra and phase of the probe and pump are shown in Fig. 2a,c, showing a linear phase over the entire spectral region, with a sharp increase in phase for the wavelengths below 450 nm of the pump pulse. This is the result of the commercially available chirped mirrors which cover a wavelength range between 450 and 950 nm. When selecting a spectral region for the excitation pulse, this pulse will therefore be close to its corresponding transform limit. The calculated transform limited pulse durations these pulses are 2.83 fs and 2.92 fs for the pump and probe respectively. After the retrieval algorithm, both probe and pump pulse durations are determined to be 3.3 fs at the sample position as shown in Fig. 2b,d. This is close to the transform limit for both beam lines and is comparable or better than high-end (N)OPA systems, capable of generating pulses within the sub-10 fs regime^18,21^. The temporal compression is achieved by using chirped mirrors alone, avoiding access to lossy or sophisticated elements such as gratings or pulse shapers.

The second important parameter to obtain a good signal to noise ratio is the shot-to-shot stability of the pump and probe. As the long-term stability of this MPC system has been shown in an earlier paper^24^, we will expand on the short-term stability. We have taken 2,000 spectra over a 2 second time window, chosen to obtain a sufficient statistical sample size whilst avoiding the effect of long-term laser fluctuations. To verify whether these acquired shots are a good representation, multiple series of shots were taken on different days for comparison. These other series showed good agreement with the results presented in this paper.

**Figure 3:**
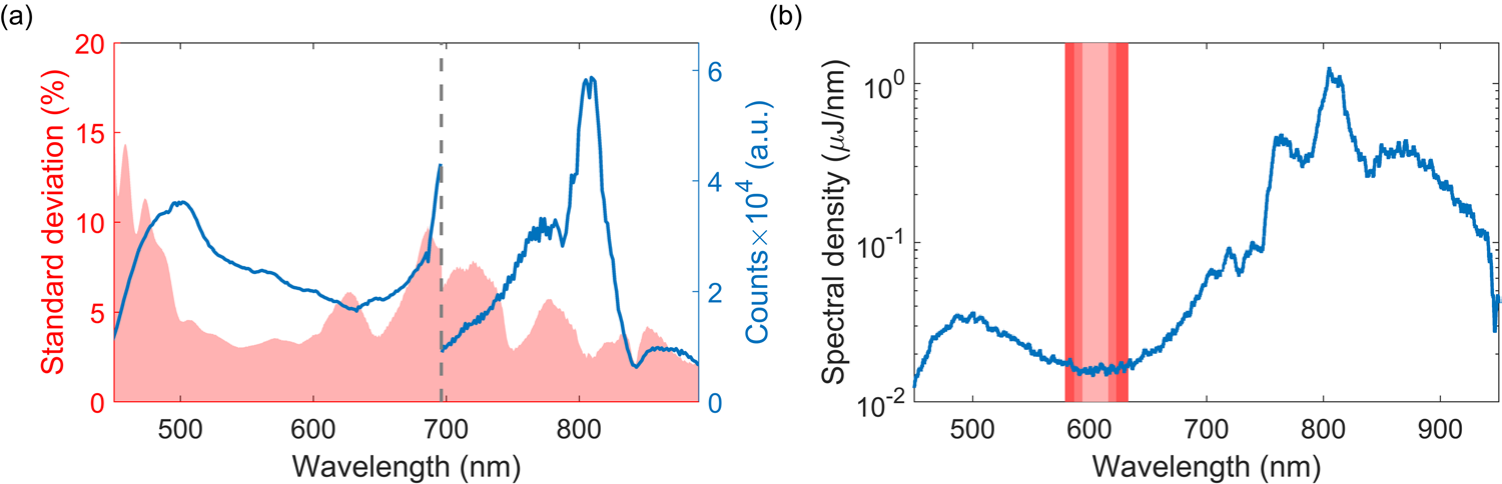
(**a**) The relative standard deviation (pink) and the averaged spectrum (blue) from 2,000 individually measured pulses generated by the MPC. The spectrum is the result of 2 separate measurements merged at 696 nm, indicated by the grey dashed line. The saturation level is at about 65,536 counts (16-bit). (**b**) The available spectral density of the pulse after the compression stage. The pink lines indicate the spectral width required for a 20 fs (dark), 30 fs (middle) and 50 fs (light) pulse duration centered at 605 nm

The averaged probe spectrum, obtained by the TAS camera, and its relative standard deviation is shown in Fig. 3a. The spectrum is cut off at 890 nm by the physical range of the detector. To prevent saturation of the camera, a combination of filters has been applied after the sample position. The shown spectrum is the result of 2 different measurements, optimized at different wavelength regions. The retrieved spectra are cut off and merged at 696 nm as indicated by the dashed grey line. A low average relative standard deviation of 4.6 % is achieved between 490 and 890 nm. Below 500 nm, the noise level increases as it lies at the edge of the spectrum. As the actual recorded spectra are continuous at the merging wavelength, no further precaution is taken around this region while calculating this. The noise profile is similar to that observed in bulk supercontinuum generation^26^. The noise characteristics originate from electrical noise, shot noise and pulse fluctuations. An estimation of the different contribution is done in the same way as Kanal et al.^27^ and is shown in Table 1. These errors are calculated from the intensity of the collected shots for the pixels corresponding to 600 nm, which relates to the low standard deviation region. The contribution from electronic noise and shot noise are an order of magnitude lower compared to the MPC supercontinuum fluctuation. Therefore, the observed standard deviation can be considered a good representation of the supercontinuum noise.

**Table 1:** Individual noise contributions of the obtained standard deviation spectrum for the pixel corresponding to 600 nm. $^*$For this wavelength, the conversion efficiency of the CMOS is 79 %

	Noise contributions at 600 nm		
	Electronic noise	Shot noise	Laser fluctuations
Value	$I_0 = 20{,}137$ counts	$N_{ph}^* = 3.11\times 10^4$	$I_0 = 20{,}137$ counts
Error	13.1 counts	$\sqrt{N_{ph}} = 176$	612 counts
Relative error	$6.51\times 10^{-4}$	$1/\sqrt{N_{ph}} = 5.67\times 10^{-3}$	$3.04 \times 10^{-2}$
Error of $\frac{\Delta T}{T}$	$9.20\times 10^{-4}$	$8.02\times 10^{-3}$	$4.30\times 10^{-2}$

Besides broadband short pulse excitation, the MPC is also an excellent candidate as a source for narrowband excitation. Previous results have shown high spectral density of the MPC over the whole visible range^22^. The narrowband excitation can be achieved by focusing the beam onto a pair of variable long-pass and short-pass filter pairs using a cylindrical lens. This allows the user to select the center wavelength and spectral width, with the edges defined as step functions, enabling the excitation to resonate with specific energy bands in materials. As for this TAS system, the available spectral density after the compression stage is shown in Fig. 3b. The spectral density is obtained by dividing the total power over the available spectrum. This shows that the minimal spectral density is 14.5 nJ/nm at 605 nm. To obtain a figure of merit, we assume narrowband excitation with a bandwidth of 22 nm around this wavelength and a sharp intensity drop off at the edges of this window, indicated by the light pink line. This results in a total pulse energy of 353 nJ. For the spot size used in this experiment (0.002 cm$^2$), it results in a maximum fluence of 175 μJ/cm$^2$. This fluence is substantially more than required for most pump-probe experiments. The 22 nm bandwidth centered at 605 nm can support a 50 fs pulse duration. The linear phase of the spectral components of the MPC pulses, as discussed previously and shown in Fig. 2a,c, show that selecting such a bandwidth will result in a pulse that is close to the transform limit of the spectrum. When considering the importance of temporal resolution, we have also calculated the spectral width of a 30 fs and 20 fs pulse at this center wavelength. These are 36 nm (middle pink) and 54 nm (dark pink) respectively (Fig. 3b). This results in a total available energy of 575 nJ and 880 nJ per pulse respectively. Furthermore, these narrow bandwidth pulses could be frequency doubled using second harmonic crystals to generate pump pulses in the UV.

**Figure 4:**
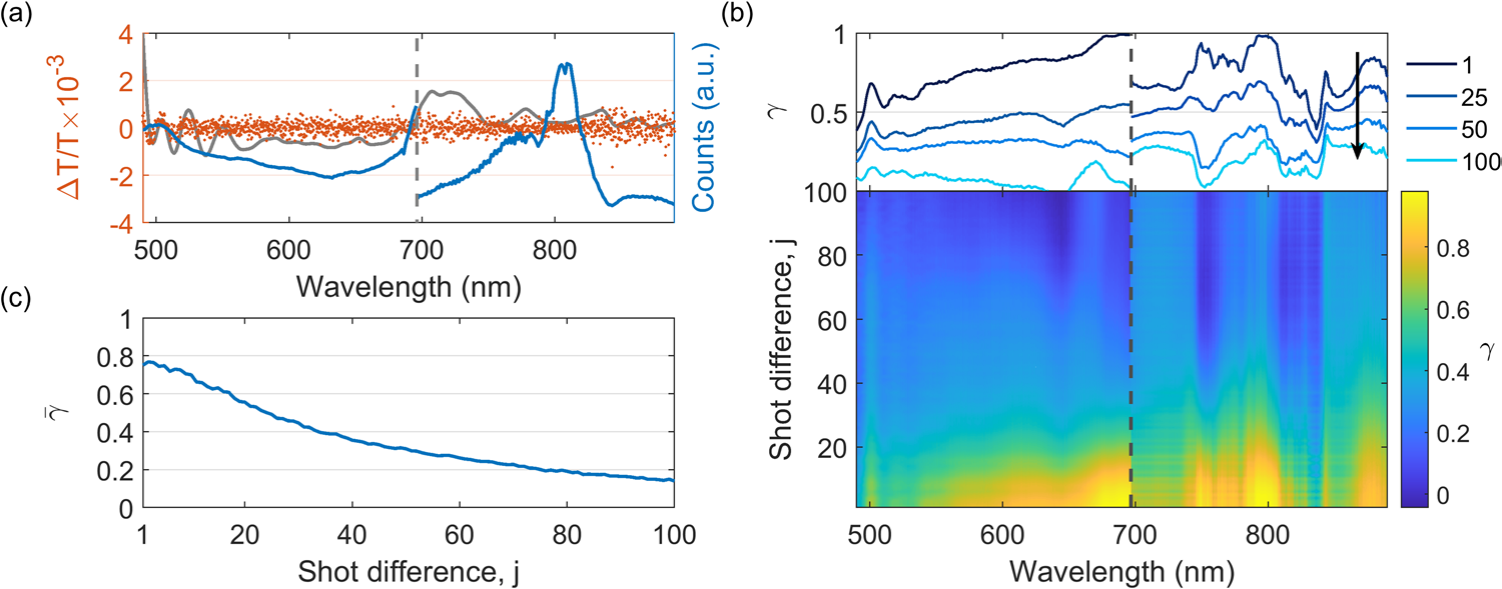
(**a**) Spectrum (blue) and $\Delta T/T$ signal (red) of 1,000 shot pairs without a sample and pump. The pseudo structures (grey) have been subtracted from the transient absorption signal. (**b**) The wavelength-dependent correlation for different shot differences, j. (**c**) Average correlation over the whole spectral range

The obtained probe spectrum between 490 and 890 nm and its corresponding transient absorption signal without a sample is shown in Fig. 4a. The transient absorption signal is obtained according to

1 $$\begin{aligned} \frac{\Delta T}{T}(\lambda , t) = \frac{T^*(\lambda , t)-T(\lambda )}{T(\lambda )}, \end{aligned}$$

where $T(\lambda )$ and $T^*(\lambda , t)$ are the ground state transmission and the excited state transmission respectively. The resulting transient absorption signal shows that, with a total of only 1,000 “on/off” pairs, a noise level of $2.6\times 10^{-4}$ RMS is achieved over the whole spectral range, which is similar to or better than other TAS systems using a bulk supercontinuum probe^28^. Significant pseudo-structures are present in the $\Delta T/T$ spectrum. These pseudo-structures (indicated in grey) are present in the same locations, but inconsistent between different data sets and are therefore subtracted by a smoothing spline (0.01) to obtain a better understanding of the base noise level of the $\Delta T/T$ spectrum. The presence of these features is the result of spectral correlation and is observed in bulk supercontinuum as well^28–30^. In future work, the effect of spectral correlation can be eliminated using probe referencing schemes, thanks to the abundance of photons in the MPC supercontinuum. Probe referencing has the added benefit of increasing the signal to noise level up to 5-fold^29^. The signal can be further improved by flattening the spectrum and using the full depth of the pixel well of the camera pixels. This can be achieved by using chromatic filters, blocking the intense fundamental region.

An ideal probe source has high shot-to-shot stability. This would result in a modulation of the probe purely from the excitation of the sample. However, the observed signal includes fluctuations of the laser condition and background noise from the camera. A good measure for the similarity of the individual shots is given by the Pearson product moment correlation coefficient^31^. The correlation coefficient is calculated according to

2 $$\begin{aligned} \gamma _{\lambda , j} = \frac{\sum ^{n-j}_{i=1}\left[ (E_{\lambda ,i}-\overline{E}_{\lambda })(E_{\lambda ,i+j}-\overline{E}_{\lambda })\right] }{\sqrt{\sum ^{n-j}_{i=1}(E_{\lambda ,i}-\overline{E}_{\lambda })^2}\sqrt{\sum ^{n-j}_{i=1}(E_{\lambda ,i+j}-\overline{E}_{\lambda })^2}}, \end{aligned}$$

where $E_{\lambda ,i}$ and $E_{\lambda ,i+j}$ is the intensity of the indicated wavelength for the *i*th shot and the $(i+j)$th shot respectively. The correlation over the spectral range of 490 nm to 890 nm is shown in Fig. 4b. A 120 Hz signal is superimposed on the correlation, caused by room lighting and is filtered out using a band-stop filter. Here, the correlation profiles for selected shot differences, j, are shown in the top panel. When comparing this to the pseudo structures in Fig. 4a, it is observed that the pseudo structures are at the same wavelengths as the areas with lowest correlation. This indicates that by eliminating the pseudo structures, via probe referencing, the overall correlation will be improved^28^. Figure 4c shows the spectrally averaged correlation coefficient over shot difference. This signal has readily been eliminated using a band-stop filter. The mean correlation between sequential shots over the whole spectral region ($\bar{\gamma _1} = 0.77$) is better than previously found in bulk supercontinuum at 1 kHz repetition rate ($\bar{\gamma _1} \approx 0.5$)^28,29^. The correlation shows a slow decay (t$_{1/2} = 35$ ms) which allows non-sequential shots to be used to calculate the transient absorption signal^27^. This is particularly interesting for high repetition rate lasers to avoid either synchronization electronics adding complexity to the system, or the use of mechanical choppers, often restricting the maximum pump modulation frequency.

### Example measurement

**Figure 5:**
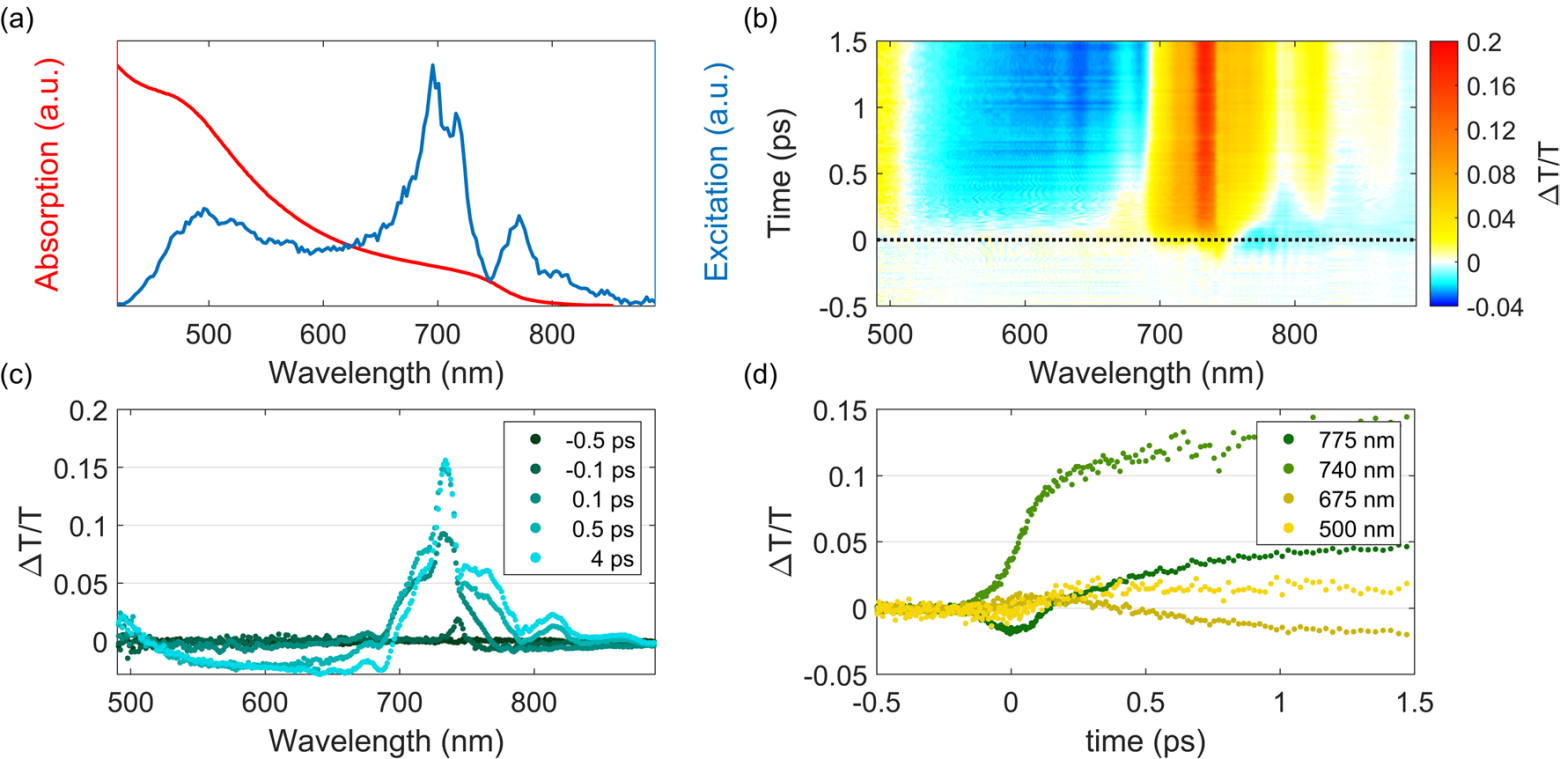
Results from a TAS experiment using 1,000 shot pairs on a spin coated MAPbI$_3$ Perovskite film under a fluence of 20 μJ/cm$^2$. (**a**) The normalized absorption spectrum of the MAPbI$_3$ sample and normalized excitation spectrum of the pump pulse. The absorption spectrum is adapted from Umari et al.^34^. (**b**) The false color plot of the TAS measurement. (**c**) Spectra at different time delays. Here, a total of 3 sequential time points around the indicated time point are averaged to obtain the time-dependent spectra and (**d**) kinetics at selected wavelengths using a 5 pixel (1.25 nm) average

As a proof of concept, we have used the MPC-TAS system to measure a spin coated methylammonium lead iodide (MAPbI$_3$) perovskite sample. This sample is placed in a vacuum chamber under dynamic vacuum to avoid photodegradation. The absorption and excitation spectra are shown in Fig. 5a. A 750 nm short-pass filter is used in the pump path to eliminate the intense MPC driving wavelengths around 790 nm. The used excitation fluence is 20 μJ/cm$^2$. A false colour plot of the transient absorption signal between 490 and 890 nm of MAPbI$_3$ perovskite is shown in Fig. 5b. No post-processing chirp correction is applied to this data, indicating the short probe pulse duration, close to its transform limit. The measured perovskite shows the typical photo-induced response as seen in previous research^32^. These signals exhibit a photobleach at 740 nm as well as the edge of a second photobleach at 500 nm, corresponding to the direct excitation of the first and second conduction band edge of MAPbI$_3$ perovskite^33^. Hot carrier cooling between 510 nm to 700 nm at early time, followed by a broad excited state absorption. And finally, the bandgap renormalization rapidly followed by state filling at 775 nm is observable. Spectral slices at fixed time points are shown on in Fig. 5c to obtain perspective on the noise level. The spectra are obtained by averaging over 1,000 “on/off” pairs per time point. The kinetics of these main features are shown in Fig. 5d.

The compressed MPC supercontinuum allows for the observation of a broad spectral range with a high temporal resolution. There are two features of the perovskite material’s excited state dynamics which can be resolved by the high temporal resolution of the TAS and high brightness of the probe. The first feature is the two-fold rise time of the main photobleach at 740 nm, which is only resolved by using ultrashort pulses. It consists of a sub-100 fs and a longer-lived rise time. These rise times have previously been assigned to carrier thermalization and hot carrier cooling respectively^35^. The second feature is a weak short-lived negative signal before a bleach signal at 500 nm. This feature arises from the bandgap renormalization of the second valence, or conduction, band and is similar to the negative signal caused by bandgap renormalization at 775 nm^33^. The high spectral density of the MPC probe over a broad spectral range allows the measurement of this feature, which is in a region of strong photoabsorption, while the short pump duration provides the temporal resolution required to observe this feature before the positive bleach takes over.

## Conclusion

In conclusion, we have demonstrated the application and capability of the MPC in the field of ultrafast spectroscopy. The generated MPC supercontinuum spans from 450 nm to 950 nm and has a high spectral density of over 14.5 nJ/nm. The high spectral density of this single light source allows the pulses to be split and used as both the probe and pump. These pulses are highly compressible, 3.3 fs pulse duration as measured using a PG-XFROG, by using chirped mirrors alone. After compression, the pulse energy is 87 μJ, resulting in a 44 % conversion efficiency. The main loss is due to the bandwidth of the chirped mirrors which can be improved in the future.

By using a line-scan camera, the shot-to-shot stability of the MPC supercontinuum is analysed. The intensity of the generated supercontinuum has an average shot-to-shot relative standard deviation of 4.6 % between 490 nm and 890 nm as measured for 2,000 pulses. By calculating the transient absorption signal from the spectrum, a $\Delta T/T$ background noise of $2.6\times 10^{-4}$ RMS is achieved by using only 1,000 shot pairs (2 second acquisition time). This can be further improved by using a probe-referencing scheme, which will also reduce the impact of spectral correlation induced pseudo-structures. A strong temporal correlation is demonstrated with a spectral average of 0.77 for sequential shots.

The results discussed in this paper paves a pathway for the MPC light source to be used in the field of ultrafast spectroscopy. The high spectral density of the MPC allows for experiments which were previously impossible. Three examples of measurements where this technique is valuable are materials with a high absorption coefficient^36^, frequency domain interferometry (FDI)^37,38^ and spatial-resolved transient absorption spectroscopy^39,40^. Highly absorbing materials are often restricted to reflection experiments as the transmitted probe is too weak to detect. By using the high intensity of the MPC supercontinuum as a probe pulse, it will be possible to observe the transmitted probe. The FDI technique requires high spectral resolution and therefore requires a high spectral density to obtain a sufficient photon count on the detector. The low spectral density of bulk supercontinuum pulses therefore limits the spectral range of FDI experiments. To perform spatial-resolved transient absorption spectroscopy, an intense probe pulse is required to observe the kinetics of a sample with a large detection area. Currently, NOPA’s, with their limited spectral range, are used to obtain the required intensities. With the MPC, similar intensities are obtained with a broader spectral range, allowing for a broader observable spectrum. The broad spectrum, short pulse duration, high spectral density and high quality beam mode can further be used in coherent multidimensional spectroscopy systems, such as 2DES, to replace often used NOPAs. Furthermore, recent developments of the MPC with high repetition lasers show great promise for MPC to expand into the field of high throughput spectroscopy^24^.

## Additional methods

### PG-XFROG

The pulse durations are obtained by a home-built PG-XFROG system. Both pump and probe are focused onto a 100 μm thick quartz slide. two polarizers are placed in the probe path in the cross-polarization configuration, one before and one after the sample. A change of polarization of the probe is obtained when the pump pulse, polarized at a 45 degree, is temporally overlapped via optical Kerr effect. The pump is delayed with respect to the probe and the transmitted probe is measured by a spectrometer (Ocean Optics HR4000) to obtain the full PG-XFROG trace. All transmissive optical components used in the TAS system have been placed in the path of the PG-XFROG probe to ensure that the measured pulse duration is the same as used in the TAS system.
